# Cytogenetic and molecular analyses of *de novo* translocation dic(9;13)(p11.2;p12) in an infertile male

**DOI:** 10.1186/1755-8166-7-14

**Published:** 2014-02-21

**Authors:** Ewa Wiland, Marta Olszewska, Andrew Georgiadis, Nataliya Huleyuk, Barbara Panasiuk, Danuta Zastavna, Svetlana A Yatsenko, Piotr Jedrzejczak, Alina T Midro, Alexander N Yatsenko, Maciej Kurpisz

**Affiliations:** 1 Institute of Human Genetics Polish Academy of Sciences, Department of Reproductive Biology and Stem Cells, Strzeszynska 32, Poznan 60-479, Poland; 2 Department of OBGYN and Reproductive Sciences, School of Medicine, University of Pittsburgh, Pittsburgh, PA 15213, USA; 3 Institute of Hereditary Pathology, Ukrainian Academy of Medical Sciences, Lysenka 31A, Lviv 79000, Ukraine; 4 Department of Clinical Genetics, Medical University Bialystok, Waszyngtona 13, Bialystok 15-089, Poland; 5 Division of Infertility and Reproductive Endocrinology, Department of Gynecology and Obstetrics, Karol Marcinkowski University of Medical Sciences, Poznan, Poland

**Keywords:** *De novo* whole arm dic(9;13)(p11.2;p12) translocation, Meiotic chromosomes, Aneuploidy rates in leukocytes, Male infertility

## Abstract

**Background:**

Whole arm t(9;13)(p11;p12) translocations are rare and have been described only a few times; all of the previously reported cases were familial.

**Results:**

We present here an infertile male carrier with a whole-arm reciprocal translocation dic(9;13)(p11.2;p12) revealed by GTG-, C-, and NOR-banding karyotypes with no mature sperm cells in his ejaculate. FISH and genome-wide 400 K CGH microarray (Agilent) analyses demonstrated a balanced chromosome complement and further characterised the abnormality as a dicentric chromosome (9;13): dic(9;13)(pter→p11.2::p12→qter),neo(9)(pter→p12→neo→p11.2). An analysis of the patient’s ejaculated cells identified immature germ cells at different phases of spermatogenesis but no mature spermatozoa. Most (82.5%) of the germ cells were recognised as spermatocytes at stage I, and the cell nuclei were most frequently found in pachytene I (41.8%). We have also undertaken FISH analysis and documented an increased rate of aneuploidy of chromosomes 15, 18, X and Y in the peripheral blood leukocytes of our patient. To study the aneuploidy risk in leukocytes, we have additionally included 9 patients with non-obstructive azoospermia with normal karyotypes.

**Conclusions:**

We propose that the azoospermia observed in the patient with the dic(9;13)(p11.2;p12) translocation was most likely a consequence of a very high proportion (90%) of association between XY bivalents and quadrivalent formations in prophase I.

## Background

Reciprocal chromosomal translocations (RCTs) are the most common structural rearrangements in humans. The incidence of RCTs is estimated at 1 in 712 live births, and the frequency at the time of prenatal diagnosis is even higher, approximately 1 in 250 pregnancies [1,2]. Most RCT's do not occur *de novo* but are inherited from one of the parents [3]. Reciprocal chromosome translocations usually have unique chromosomal breakpoints. During meiosis, the rearranged chromosomes produce a quadrivalent configuration leading to chromosomally unbalanced gametes. The pattern of chromosome segregation that can occur in the meiotic cells of a RCT carrier depends greatly on the chromosomes involved, the location and number of the breakpoints, and the size of the imbalance [4-7]. However, the roles of the meiotic segregation mechanisms are not yet fully understood. No universal rules can be applied to the majority of balanced translocation carriers; therefore, each case must be considered to be unique [8,9].

Heterozygous RCT carriers commonly have normal semen analysis, histological composition of seminiferous tubules, gametogenic differentiation, sperm counts, sperm morphology and sperm motility. However, in some cases, chromosomal translocations may cause a complete spermatogenic arrest. The presence of translocation breakpoints in close proximity to the centromere and the involvement of acrocentric chromosomes are associated with the most destructive effects on spermatogenesis, resulting in azoospermia or severe oligozoospermia. In RCT carriers, azoospermia is often due to an arrest at the first meiotic division of gametogenesis [10]. Individuals with RCTs involving the X chromosome commonly suffer from infertility. In males with Y chromosome translocations, infertility may depend on the physical locations of the translocation breakpoints [11]. The question of why some translocation carriers suffer from male infertility and others do not is still unresolved [9].

We describe here a case of a non-obstructive azoospermic male carrier of a *de novo* reciprocally balanced translocation resulting in dic(9;13)(p11.2;p12). This type of rearrangement belongs to the class of whole-arm translocations, which result from the centromeric fusion of two chromosomes [12]. In this type of translocation, one possibility is that both breakpoints are located juxta-centromeric and both derivative chromosomes contain their own centromere sequences. Another mechanism is when one breakpoint occurs within the centromere and the other is located juxta-centromeric; one derivative chromosome therefore has a hybrid centromere and the other one has a reduced but conserved original centromere [13]. Whole arm translocations occur most frequently between chromosomes with similar alphoid domains [12]. However, carriers of a (9;13)(p11;p12) translocation have been described only a few times, and all of these cases were familial [12,14]. In contrast to the previously described cases, the patient described herein with dic(9;13)(p11.2;p12) has an increased number of immature germ cells but no mature sperm cells in his ejaculate. This enabled us to perform cytogenetic analysis of the chromosomes of numerous different spermatogenic cells.

The literature indicates the possibility of altered genetic control over not only meiotic but also mitotic cell divisions in infertile males [15,16]. This suggestion stems from the fact that predispositions to the nondisjunction of meiotic chromosomes with concomitant observations concerning the mitotic chromosomes of somatic cells were well documented [15,16]. Furthermore, it has been reported that karyotypically normal patients with non-obstructive azoospermia have an increased mitotic instability in germ cells and/or somatic tissues [17-21]. Therefore, here we have undertaken a study to evaluate if our azoospermic patient, a carrier of dic(9;13)(p11.2;p12), demonstrates increased aneuploidy rates for chromosomes 15, 18, X and Y in his peripheral blood leukocytes. An additional nine patients with non-obstructive azoospermia and normal karyotypes and seven fertile normozoospermic men were included in the study. We chose to study chromosomes 15, 18, X and Y because there are reports indicating a high aneuploidy rate of these particular chromosomes (and especially the sex chromosomes) in different cohorts of infertile men [15,16,21,22].

## Results

**Figure 1:**
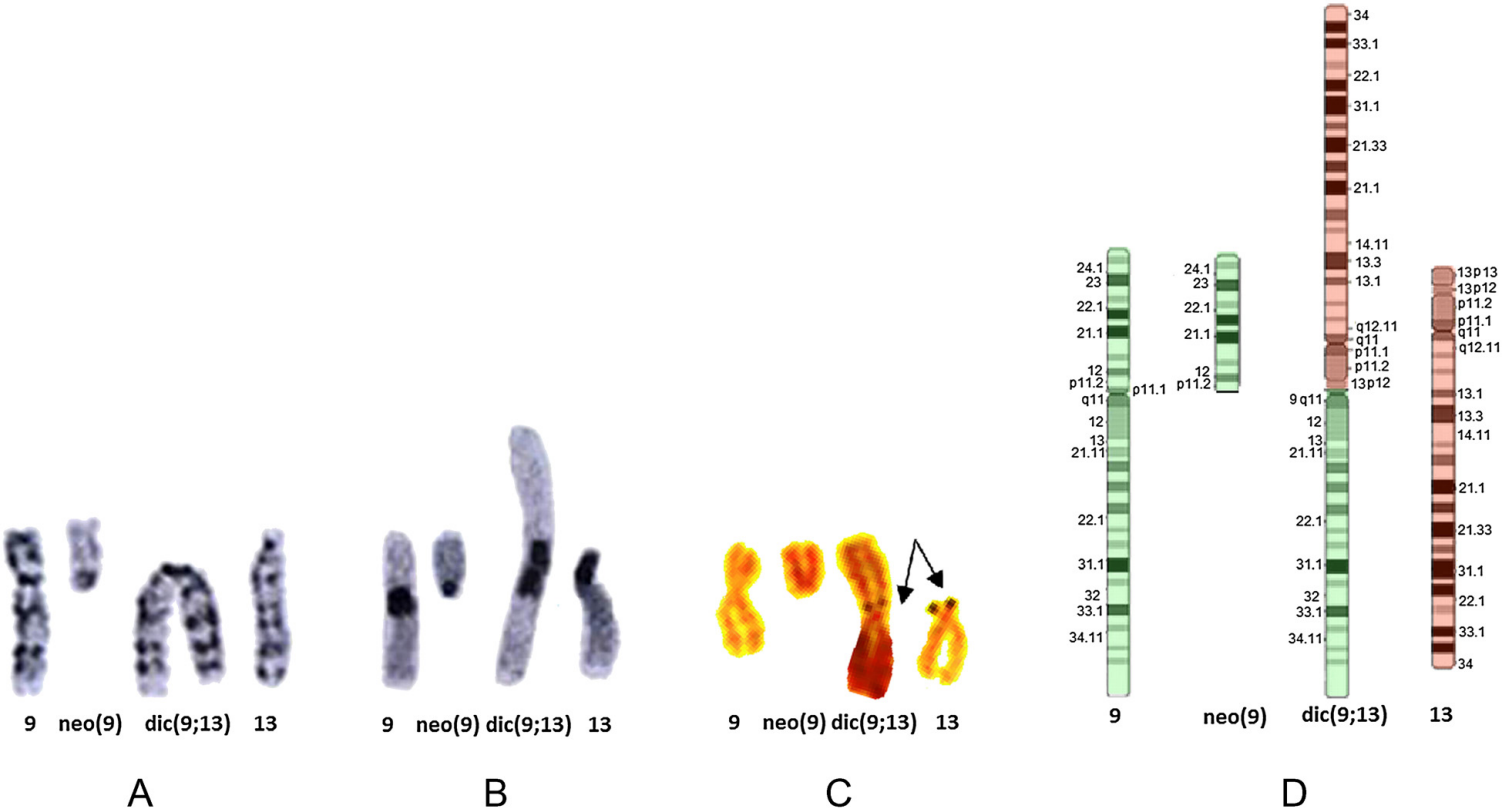
**Conventional cytogenetic analyses.** Chromosomes involved in the dic(9;13)(p11.2;p12) translocation. **A**. G-banded chromosomes, **B**. C-banded chromosomes, **C**. NOR-banded chromosomes; the satellites (indicated with arrows) are present on both the normal and dic(9;13) chromosomes, **D**. Ideogrames of G-banded chromosomes involved in this translocation.

Conventional cytogenetic analysis (GTG bands, C-bands and NORs) of an infertile patient revealed a 46,XY,t(9;13)(p11;p12) karyotype (Figure 1A-C). Microdeletions in the AZF regions and mutations in the SRY and CFTR genes were not found. Because the parents of our patient had normal karyotypes, we concluded this was a case of *de novo* translocation.

**Figure 2:**
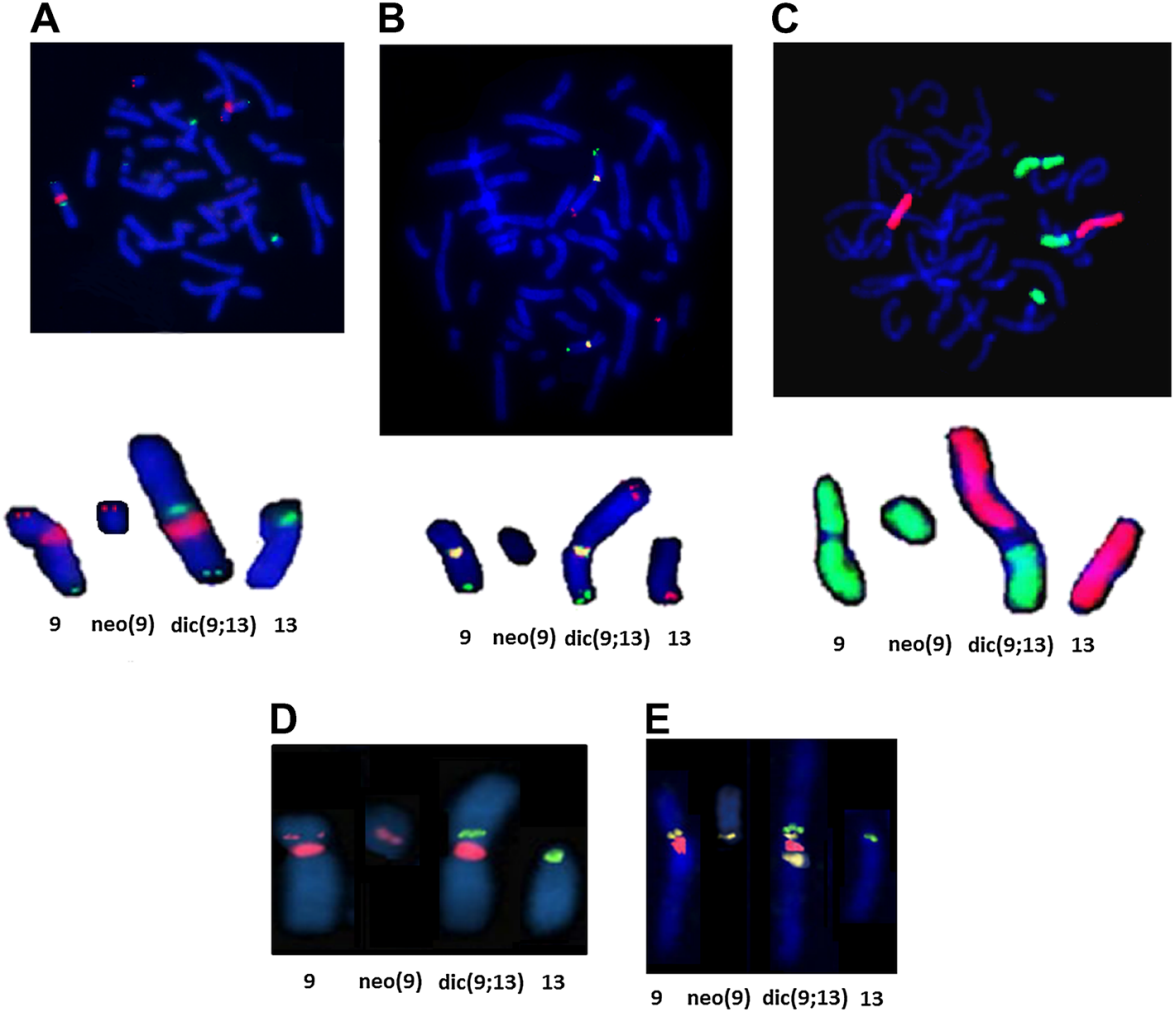
**FISH analysis.** The chromosomes involved in the dic(9;13)(pter→p11.2::p12→qter),neo(9)(pter→p12→neo→p11.2) translocation: examples of FISH results (details in Additional file 1: Table S1). **A**. FISH results for 4 probes: centromere 9 probe (red), 9p subtelomere (red), 9q subtelomere (green) and centromere 13/21 (green). **B**. FISH results for 3 probes: centromere 9 (yellow), 9q subtelomere (green) and 13q subtelomere (red). **C**. FISH results for painting probes: 9 wcp (green) and 13 wcp (red). **D**. FISH results for 3 probes: 9 centromere (red), 9p11.2 - specific RP11-259A5 (red) and 13/21 centromere probe (green) showing that chromosome 9 region 9p11.2 is present on the normal and dic(9;13) chromosomes. **E**. Tricolour FISH results with chromosome 9 (red) and 13/21 (green) centromeric probes shows the presence of these probes on dic(9;13) and each of the normal chromosomes. The yellow probe RP11-318 K12 corresponds to the 9q13 region and is present on the dic(9;13) chromosome and the normal chromosome 9q12.

Based on conventional analysis, we expected that the dic(9;13)(p11;p12) chromosome possessed two centromeres (one from chromosome 9 and the second from chromosome 13) and the satellite sequence of chromosome 13p12 (Figure 1D). To confirm this suggestion, FISH analysis was performed with various probes (listed in Additional file 1: Table S1). The positions of the applied probes and the FISH results are shown in Figure 2A-E. As can be seen in Figure 2A, D and E, dic(9;13) contains both centromere 9 and centromere 13 sequences. This chromosome also revealed signals for both the 9q pericentromeric (which is expected) and 9p pericentromeric probes. Interestingly, the 9p11.2 pericentromeric probe showed multiple signals on dic(9;13); one was located between centromere 9 and centromere 13, and one was on 9q proximal to centromere 9 and also on neo(9) (Figure 2E). Because C-bands (Figure 1B) did not indicate the presence of heteromorphic inv(9)(p11q12) on the chromosome 9 involved in the translocation t(9;13)(p11;p12), we excluded this variant. The signal on 9q was most likely the result of cross-hybridisation. Analogously, cross-hybridisation FISH signal from satellite III DNA were present on 13 and 21 chromosomes (Figure 2A).

The short chromosome neo(9) segregated with the normal chromosome13 and der(9;13) and was present in all analysed metaphases. Neo(9) did not show signals from centromere 9 and centromere 13 but did show a signal from 9p (9p11.2) (Figure 2D and E). This probe contains a repetitive sequence that is most likely more similar to that of satellite I, which evolutionary was a part of the centromere sequence in primates. As a consequence, we suggest that neo(9) most likely has a neocentromere and can be described as neo →9p11.2. Accordingly, the full karyotype was interpreted as 46,XY,dic(9;13)(pter→p11.2::p12→qter),neo(9)(pter→p12→neo→p11.2).

### Array CGH results

To assess whether the azoospermic patient dic(9;13)(p11.2;p12) has a balanced translocation and to test for potential genomic micro-aberrations, we performed a genome-wide analysis using the 400 K CGH array (Agilent Technologies, Santa Clara, CA,USA). We identified 26 genomic copy number variations (CNVs) that showed a loss or gain of genomic DNA in the patient vs. control male DNA (Additional file 2: Table S2). Special attention was given to imbalances that were mapped to the genomic regions surrounding the anticipated chromosome translocation breakpoints. However, we did not identify any detectable gain or loss on the regions of chromosome 9 and 13 involved in the translocation. Our CGH analysis revealed that most of the genomic variants are polymorphic, because they were found with relatively high frequencies in the normal population in the Toronto DGV database (CNVs).

### Analysis of ejaculated meiotic cells

**Figure 3:**
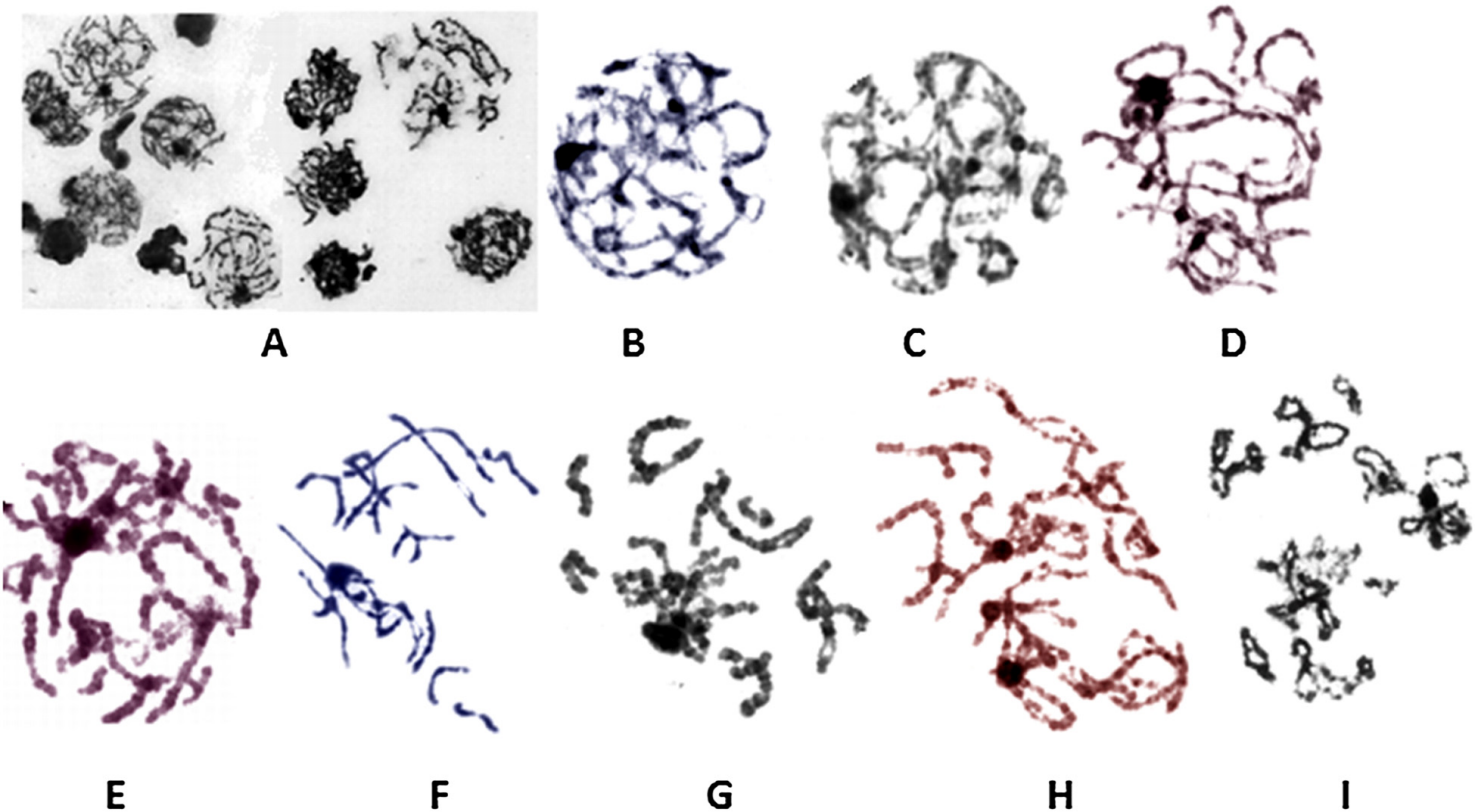
**Meiotic chromosomes. A**. View of the spermatocytes found in the ejaculate of the azoospermic carrier of the dic(9;13)(p11.2;p12) translocation observed using a light microscope after Giemsa staining. **B-I**. Examples of chromosomes in different stages of meiotic pachytene found in the ejaculate of the carrier: **B**. Zygotene. **C**. Late zygotene. **D**. Early pachytene. **E**. Middle pachytene. **F**. Late pachytene. **G**. Early diplotene. **H**. Diplotene. **I**. Late diakinesis.

Gametogenic cells at different phases of differentiation were found in the ejaculate of the dic(9;13)(p11.2;p12) carrier (Figure 3A-I). The percentage of cell nuclei identified in the ejaculated sample is shown in Table 1. Most of the cells were recognised as spermatocytes (82.5%), most frequently at prophase I stage (85.5%). Normal spermatozoa were not found. Simultaneously, 7% of the revealed cell types were leukocytes. The proportion of nuclei during the subsequent steps of prophase I is shown in Table 2. Most of the nuclei were in the leptotene stage (29.8%) and in pachytene (41.8%). Asynapsed regions of chromosomal fragments not involved in the quadrivalent complexes were present in over 80% of the pachytene nuclei (Figure 3F and G).

**Table 1:** Proportion of different types of cells found in the ejaculate of the azoospermic carrier of dic(9;13)(p11.2;p12)

**Cells recognized in ejaculate**								
**Spermatocytes 82.5%**				**the other cells 17.5%**				
**Prophase I**	**Metaphase I**	**Anaphase I**	**Telophase I**	**Spermatid I**	**Spermatid II**	**Amorphic spermatozoa with flagella**	**Leukocytes**	**Not identified**
85.5%	0.8%	0.2%	6.7%	6.o%	0.3%	0.5%	7%	3.7%

**Table 2:** Proportion of different stages of prophase I in the exfoliated spermatocytes found in the ejaculate of the azoospermic carrier of dic(9;13)(p11.2;p12)

**Leptotene**			**Zygotene**		**Zygotene/pachytene**	**Pachytene**			**Diplotene**	**diakinese**
29.8%			10.5%		16.6%	41.8%			0.7%	0.6%
early 7.9%	middle 70.4%	late 21.7%	early 28.3%	middle 55.1%		early 46%	middle 1%	late 53%		

A depiction of the most likely quadrivalent figure in the meiotic pachytene stage consisting of the chromosomes involved in the dic(9;13)(p11.2;p12) translocation is shown in Additional file 3: Figure S1.

**Figure 4:**
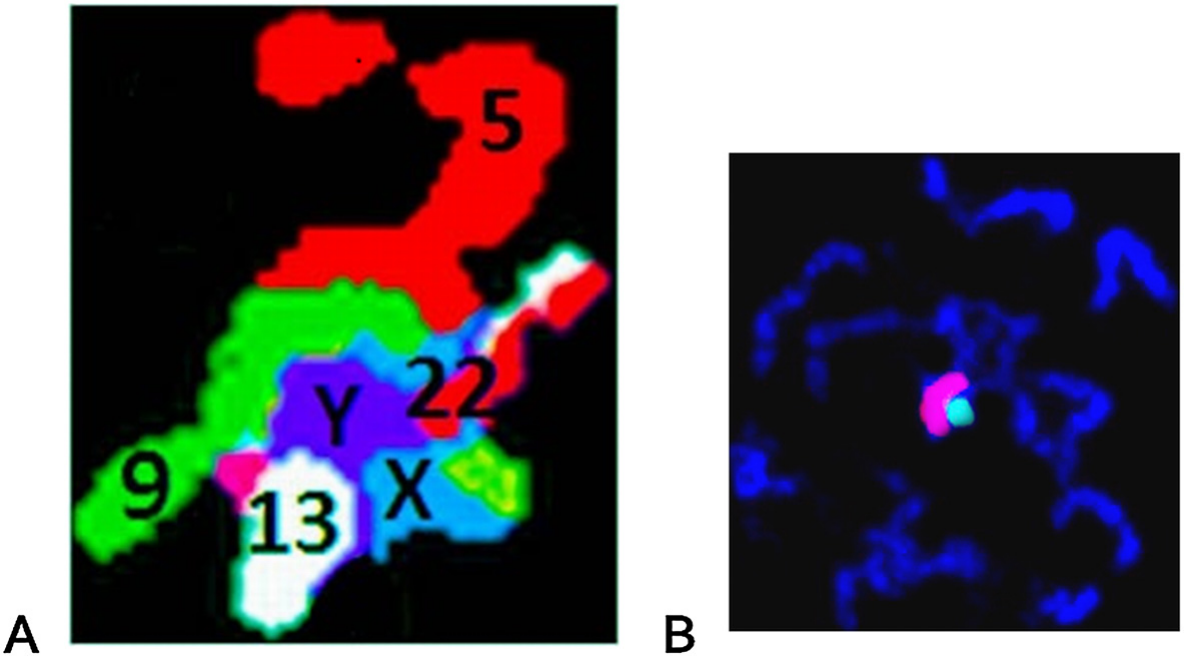
**XY FISH signals in pachytene.** FISH on prophase I chromosomes from the exfoliated spermatocytes found in the ejaculate of an azoospermic carrier of dic(9;13)(p11.2;p12). **A**. Example of an association of chromosomes involved in the dic(9;13)(p11.2;p12) translocation with an XY bivalent and additional chromosomes 5 and 22 (MultiFISH signals, false colours) (MetaSystems). **B**. Bi-colour FISH signals with paint probes showing the XY bivalent in meiotic pachytene (red probe = X chromosome; green = chromosome) (the FISH probes are described in Additional file 1: Table S1).

In 90% of the pachytene nuclei, XY chromosome bivalents were found in complex with the quadrivalents of the chromosomes involved in the dic(9;13)(p11.2;12) translocation (Figure 4A and B). In all the pachytene quadrivalents, two or more additional somatic chromosomes were also associated with these structures (we observed chromosomes 5 and 22 very frequently) (Figure 4A). In 1.4% of the pachytene nuclei, XY bivalents were not associated with the quadrivalents, and in 8.6% of the pachytene nuclei, we observed univalents of the X and Y chromosomes. About half of the spermatids/amorphic spermatozoa had FISH signals from the X chromosome, and half had signals from the Y chromosome. Approximately 0.74% of the spermatids did not reveal an X or Y FISH signal. XX and YY FISH signals were detected in 0.1% and 0.15% of the spermatids, respectively. These frequencies were similar to the results obtained from the sperm cells from our normozoospermic control group (C) (n = 7, data published by Wiland, 2010) [23].

### Aneuploidy analysis of somatic peripheral leukocytes

The results of analysis of chromosomes hyperhaploidy 15, 18, X and Y in nuclei of leukocytes from peripheral blood of the dic(9;13)(p11.2;p12) carrier and the control donors (C) and non-obstructive azoospermic patients (A) (n = 9) are reported in Table 3.

**Table 3:** Frequency of hyperhaploidy of chromosomes 15, 18, X and Y in the nuclei of leukocytes from the azoospermic carrier of dic(9;13)(p11.2;p12), the group of nine patients with azoospermia (A), and the mean control values obtained for seven fertile donors with normozoospermia (C)

**Karyotype**	47,+15	47,+18	47,+X	47,+Y	48,+XY	Polyploidy 4n
**Carrier of dic(9;13)**	0.37^	0.29^	0.85^▾	1.15^▾	0.02	0.13^
**Patients with azoospermia (A)**						
**No. 1**	0.17	0.18	0.25	0.04	0.01	0.04
**No. 2**	0.11	0.19	0.32	0.29	0.01	0.04
**No. 3**	0.28	0.20	0.50	0.04	0.01	0.07
**No. 4**	0.09	0.26	0.10	0.10	0.01	0.11
**No. 5**	0.06	0.06	0.20	0.07	0.01	0.06
**No. 6**	0.42	1.42	0.74	0.42	0.07	0.28
**No. 7**	0.22	0.22	0.43	0.17	0.03	0.20
**No. 8**	0.42	0.15	0.47	0.25	0.03	0.19
**No. 9**	0.42	0.38	0.51	0.51	0.03	0.09
**Mean value (A) *± SD***	0.24^ *± 0.15*	0.34^ *± 0.42*	0.39^ *± 0.20*	0.21^ *± 0.18*	0.02 ± *0.02*	0.12^ ± *0.08*
**Control group (n=7)**						
**Mean value (C) *± SD***	0.08 *± 0.04*	0.06 *± 0.06*	0,11 *± 0.07*	0.08 *± 0.06*	0.01 ± *0.01*	0.04 *± 0.03*
**Range**	0.04 – 0.14	0.01 – 0.16	0.04 – 0.25	0.04 – 0.20	0.00 – 0.02	0.01 – 0.09

^values significantly higher from mean control value C [p<0.05].

▾values were significantly higher from mean control value A [p<0.05].

In the leukocytes of nine patients with azoospermia (Table 3), the mean value (A) of the hyperhaploidy of individual chromosomes 15, 18, X and Y ranged from 0.21% to 0.39%. The mean frequencies of leukocytes with a karyotype of 48,XXYY were 0.02%, and the frequency of leukocytes with polyploidy (4n) was 0.12% (Table 3). With the exception of the mean frequency of leukocytes with the karyotype 48,XXYY, the other mean values (A) were significantly higher when compared to mean values obtained for the control group (C) (Table 3).

In the leukocytes of control males (C) (n = 7), the mean frequencies of hyperhaploidy of the individual chromosomes 15, 18, X and Y ranged from 0.06% to 0.11%. The mean frequency of leukocytes with the karyotype 48,XXYY was 0.01% and the frequency of leukocytes with polyploidy (4n) was 0.04% (Table 3).

In the leukocytes of the dic(9;13)(p11.2;p12) carrier, the frequency of the hyperhaploidy of individual chromosomes 15, 18, X and Y ranged from 0,29% to 1.15%. The frequency of leukocytes with the karyotype 48,XXYY was 0.02%, and the frequency of leukocytes with polyploidy was 0.13% (Table 3).

Similar to results of the group of the azoospermic patients (A) (with the exception of the mean value of the frequency of leukocytes with the karyotype 48,XXYY), the other mean values (A) obtained for hyperhaploidy were statistically higher than the mean values in control group (C) (Table 3). Moreover, the frequencies of leukocytes with hyperhaploidy 47,+Y and 47,+X were statistically higher also in comparison with mean values (A) obtained for the azoospermic group (Table 3).

## Discussion

We herein report an intriguing case of a *de novo* whole-arm translocation 46,XY,dic(9;13)(pter→p11.2::p12→qter),der(9)(pter→p12→neo→p11.2) in an infertile male with high number of germ meiotic cells but no spermatozoa in his ejaculate. This phenomenon prompted us to perform an analysis of the meiotic chromosomes in the numerous ejaculated gametogenic cells. We found a large number (82.5%) of spermatocytes and only 6.3% of spermatids, indicating that spermatogenesis could proceed to the spermatid stage, but not efficiently. In previous studies of the frequencies of different spermatogenic stages of prophase I in normal spermatogenesis, 1% - 7% - 9.5% of the cells were in leptotene, 0.5% - 4% - 0.8% were in zygotene, and 98% - 88% - 89.7% were in pachytene [24-26]. We observed a high proportion of cell nuclei at the pachytene stage (41.8%) (of these, 53% were in late pachytene) (Table 2) in our patient; this may indicate that spermatogenesis was not seriously disrupted until diplotene. This may further suggest the presence of a mechanism arresting meiotic progression at the end of the pachytene stage. At the same time, however, the high proportion of cells in leptotene (29.8%) (Table 2) may indicate that the first breakdown of the meiotic process occurred very early. According to the literature, meioses in males with non-obstructive azoospermia are very heterogeneous. In 5% of cases, the cell divisions approached an arrest at zygotene; in approximately half of the cases, the cells were arrested in the pachytene stage, and in approximately 45% of the cases, there were no meiotic cells observed. Among the males with non-obstructive azoospermia in whom cells in pachytene stage were present, 70% revealed alterations in synaptonemal complexes [10]. Given the potential reasons for lack of spermatozoa in our patient, we took into account a possibility that the breakpoints in chromosomes involved in the dic(9;13)(p11.2;p12) translocation may have altered the expression of autosomal genes that are critical for meiosis and located at a region involved in breakpoints [27,28]. However, this hypothesis is unlikely to be true, because we observed a whole-arm translocation with breaks in centromeric regions comprised of non-coding and non-transcribed α-satellite sequences. Therefore, we propose that a key factor triggering the arrest of meiosis is a pachytene configuration with the involvement of acrocentric chromosome 13, dic(9;13) and neo(9) with a likely terminal asynaptic segment (Additional file 3: Figure S1). The presence of acrocentric chromosomes in a translocation has been thought to be more deleterious for fertility than translocations not involving acrocentric chromosomes [29-31]. Unfortunately, in the case of the dic(9;13)(p11.2;p12) carrier, we could not analyse quadrivalents in synaptonemal complexes, because the remaining aliquot of ejaculate was fixed in Carnoy's fixative and therefore not suitable for further analysis. We could only search for asynapsed regions in the well-visible chromosomes, which were not associated with quadrivalents (Figure 3F and G.). We observed asynapsed regions in different chromosomes in over 80% of the pachytene nuclei. Simultaneously, almost all (90%) of the quadrivalents in pachytene involved in the translocation were associated with XY bivalents. Similarly, a high frequency of association between pachytene quadrivalents and XY chromosomes was also detected in other translocation carriers, for example in 96% of azoospermic t(1;21) carriers [32]. The association between XY bivalents and the asynapsed regions of chromosomes during pachytene is a well-known phenomenon [28,30,31,33,34]. These associations have been reported in more than 30 RCT carriers and associations were noted in over 90% of the cases. It has even been suggested that the asynapsed regions occurring in quadrivalents may attempt to pair with the XY bivalent to avoid detection by the pachytene checkpoint, which monitors synapsis phenomenon [30,31]. Different hypotheses have been proposed to explain the meiotic arrest as a consequence of the quadrivalent-XY bivalent association [35-37] but the currently prevailing hypothesis that pachytene asynapsis drives the inactivation of the meiotic X and Y chromosomes and leads to substantial postmeiotic repression in spermatids [38]. In the case of this dic(9;13)(p11.2;p12) translocation, the structure containing the translocated chromosomes and the XY bivalent in pachytene quadrivalents was joined by two or more additional somatic chromosomes (Figure 4A). This may suggest that this association was a non-specific one, but that it can arise from the fact that a noncentromeric heterochromatin block in 9q and the heterochromatin of acrocentric chromosomes are especially prone to synaptic abnormalities (breaks and unsynapsed regions) [33].

Recent reports indicated that the vast number of infertile patients, particularly with non-obstructive azoospermia, have an elevated level of aneuploidy in their somatic cells (peripheral leukocytes), which may be associated with instability of the mechanisms controlling cell divisions. For instance, interference with the cellular organisation of the microtubule system and kinetochores could lead to errors during chromosome segregation in both meiotic and mitotic cell divisions [17-20]. The results indicated an association between the non-disjunction of mitotic chromosomes in somatic cells (leukocytes of peripheral blood) and the non-disjunction of meiotic chromosomes in infertile males and were first reported by Gazvani et al [15,16]. Evidence supporting the phenomenon that patients with impaired spermatogenesis may have a predisposition for chromosome non-disjunction in somatic cells was also presented by De Palma and co-workers [22]. A study of leukocytes from patients with azoospermia reported a normal conventional karyotype in somatic cells, but showed a several-fold increase in the aneuploidy of chromosomes X and Y, suggesting an increased chromosome aneuploidy [21,22]. To test this hypothesis, we have undertaken here an evaluation of whether the azoospermic dic(9;13)(p11.2;p12) carrier and the selected patients with non-obstructive azoospermia (A) and normal conventional karyotype (C) revealed an increased rate of somatic cell aneuploidy (Table 3). Based on the results obtained (Table 3), we can confirm the data that have been previously reported [21,22]. We also observed that in patients with azoospermia, the mean frequency of the karyotypes 47,XXY and 47,XYY and aneuploidy of chromosomes 15 and 18 in leukocytes were significantly higher than in controls (Table 3). The highest frequency of hyperhaploidy was found in the leukocytes of the dic(9;13)(p11.2;p12) carrier and affected the X and Y chromosomes. It was suggested that malsegregation affects the various chromosomes differently, and the sex chromosomes appear to be more at risk of partition errors than the autosomes [16,22]. At the same time, it is known that cells with additional X and Y chromosomes maintain the ability to divide meiotically [39,40]. It should also be noted that in some males with normozoospermia, an elevated aneuploidy level in leukocytes can also be observed, paralleling the elevated aneuploidy rate in spermatozoa (this phenomenon is known as “stable variants” of aneuploidy) [41].

In the case of the azoospermic dic(9;13)(p11.2;p12) carrier, a very high proportion (90%) of prophase I quadrivalents were associated with XY-bivalents, and this seems to be the critical factor for the observed azoospermia. We speculate that in this case, a mitotic instability could be an additional co-founding factor leading to spermatogenetic arrest. Earlier described cases of t(9;13)(p11;p12) were familial and therefore did not result in azoospermia [12,14].

The spermatological phenomenon of the increased exfoliation of immature germ cells is well known in patients with spermatogenic failure, but the pathogenetic reasons for this are still unclear [42]. It is reasonable to expect that this phenomenon will have a complex heterogeneous nature. In groups of patients with astheno-terato-oligozoospermia the exfoliation concerned mainly cells from the adluminal cell compartment of the seminiferous tubules (spermatocytes and spermatids) [43]. The question is whether the increased number of immature germ cells represents a specific alteration of the sperm cell precursors. Further questions can be raised because in about half of the cases with increased exfoliation, urogenital inflammation can occur simultaneously [44]. It is known that the increased expression of genes involved in the inflammatory-like response can also be observed in azoospermic men without seminal infections [45]. In smears from ejaculated cell pellets in our azoospermic dic(9;13)(p11.2;p12) carrier, leukocytes could be observed (approximately 7%), which could suggest the remnants of a past inflammatory reaction (the microbiological analysis of this ejaculate was negative). Therefore, we cannot ignore the abundance of leukocytes, because only singular leukocytes occur in normal sterile ejaculate. Thus, similar to other azoospermic cases, it can be proposed that the observed high number of immature germ cells in the ejaculate from our patient may be a consequence of rather than a cause of azoospermia [42]. It is also known that the disruptive effects of environmental toxicants on cell junctions are mediated by non-receptor tyrosine kinases (e.g., c-Src and FAK) and cytokines provoking oxidative stress because such damage is often observed after low-level exposure before apoptosis occurs [46-48]. Significantly, these signalling pathways converge on polarity proteins that regulate intercellular junctions.

Genetic counselling was offered to the carrier and his wife. The probability rate for unbalanced progeny with trisomy/monosomy of the 9p11.2 segment after adjacent-1 segregation at birth for paternal carriers was estimated at 11.8%, and the odds of unbalanced progeny at prenatal diagnosis was 57% [49]. The probability of natural conception was very low, but an option for ICSI after testicular biopsy and pre-implantation genetic diagnosis was discussed with the couple because the presence of immature germ cells in the ejaculate could indicate spermatogenic activity in the testes [50]. Unfortunately, bilateral testicular biopsies did not reveal any sperm cells (N. Huleyuk, personal communication). As a result, fertilisation with a donor’s sperm was proposed, which resulted in the birth of a child.

## Conclusions

We propose that the most likely reason for the development of the azoospermia in the patient with dic(9;13)(p11.2;p12) was a very high proportion (90%) of an association between XY bivalents and quadrivalent formations in prophase I. We propose that instability in chromosome segregation during consecutive cell divisions might be a contributing factor inducing spermatogenic disruption in the case of this azoospermic patient, although it is difficult to define the strength of this effect.

## Methods

A 34-year-old man revealed a 3-year history of infertility and had no ejaculated spermatozoa in two semen analyses 3 months apart. The patient’s physical examination did not reveal any constitutional abnormalities or obstruction defects in the reproductive tract. The patient and the individuals in the control groups provided written consent to be included in the study. The Local Bioethical Committee of the Medical University of Poznan granted permission for this study.

### Conventional cytogenetic studies

Cytogenetic studies were performed on the chromosomes from *in vitro* cultured peripheral blood lymphocytes of the patient and his parents according to standard procedures. The interpretation of the breakpoints position on the chromosomes was based on the classic GTG banding technique with a resolution of 550 bands/haploid set [51]. Thirty metaphase cells were scored. In all cells, a translocation between chromosomes 9 and 13 was found. To further characterise this abnormality, the C- and NOR-banding techniques and fluorescent *in situ* hybridisation (FISH) mapping with different probes for chromosomes 9 and 13 were applied.

### Fluorescence *in situ* hybridisation (FISH) analysis

FISH analysis was carried out on metaphase and interphase cells from the peripheral lymphocyte cultures and germ cells isolated from the patient’s ejaculate. Bi- or tricolour FISH experiments were performed using different combinations of probes directly labelled with either Spectrum Orange-dUTP or Spectrum Green-dUTP (Abbot Molecular/Vysis Inc, Des Plaines, IL), obtained from Cytocell Technologies Ltd. (Cambridge, UK). BAC (bacterial artificial chromosomes) probes were selected from the Genome Browser database (http://genome.ucsc.edu/) based on their physical map location (near the centromeres) for chromosomes 9 and 13/21, locus-specific chromosome 9 and 13 probes, and chromosomes 6, 14, 15, 18, X and Y. In addition, we used alpha-satellite centromeric, subtelomeric or whole chromosome painting probes (Additional file 1: Table S1). To identify the chromosomes forming complexes in pachytene, we also used MultiFISH probes (MetaSystems, Altussheim, Germany). A list of the probes used and the appropriate descriptions are presented in Additional file 1: Table S1.

FISH analyses with direct label probes were performed according to the manufacturer’s protocols. The hybridisation mixture (3 μl of each probe plus hybridisation buffer) was applied to each slide. A tricolour FISH was performed, where the combination of mixed (1:1) green- and red-labelled probes on the same centromere yielded a yellow signal. The probes were covered with 20×40 mm coverslips and sealed with rubber cement. The probes and cellular DNA were denatured for 2 min at 75°C. Hybridisation was carried out overnight in a humidified chamber at 37°C. After hybridisation, the slides were washed for 2 min in a solution of 0.4× SCC at 72°C and then for 30 sec in a solution of 2× SCC/0.5% Tween-20. Counterstaining was performed with DAPI/anti-fade reagent.

DNA from the RPCI-11 BAC library (Invitrogen/Life Technology, Inc.) was used for characterisation of the dic(9;13) breakpoints. BAC clones were cultured in LB broth for 17 hours at 37°C, and DNA was isolated using a QIAprep Spin Miniprep kit (Qiagen). Three micrograms of DNA were labelled using a Nick Translation kit (Abbott, Des Planes, IL) according to the manufacturer’s protocol. The hybridisations and washing procedures were performed using the same conditions as described above. Counterstaining was performed with DAPI/antifade reagent.

The hybridisation signals were observed using an Olympus Bx41 microscope fitted with a triple-band-pass filter for DAPI/FITC/Rhodamine and an oil-immersion 100 × objective with a 1.25 NA. The efficiency of FISH performed with direct label probes both the mitotic and meiotic cells was at least 98%.

### Aneuploidy analysis of peripheral leukocytes chromosomes

Overall, 17 males were enrolled to this study, including one dic(9;13)(p11;p12) carrier, nine patients with non-obstructive azoospermia (Nos. 1-9, Table 3), and seven control male donors (age 23-30 years) with normozoospermia, proven fertility and a normal karyotype [52]. In patients 1-8, histological analyses of testis biopsies showed spermatogenetic arrest at spermatocyte stage II, while patient 9 exhibited no meiotic cells (hyalinisation of the basement membranes of seminiferous tubules). All the patients had normal karyotypes (46,XY) and were negative for AZF deletions. The aneuploidy level of chromosomes 15, 18, X and Y were tested in the interphase nuclei of peripheral blood leukocytes culture using tricolour FISH. A total of 5,000 cells were scored for each case. The results are presented as means ± standard deviation (SD) throughout the study. The data were analysed using Fisher’s exact test with α = 0.05. A statistically significant difference was accepted when the *P*-value was <0.05.

### Meiotic cell collection and preparation

We obtained two ejaculate samples from our patient at a three-month interval. The ejaculates were collected by masturbation after 5 days of sexual abstinence. After liquefaction at room temperature, a seminological analysis was performed, and the different cells were preliminarily identified as meiotic cells. The sample aliquots were then washed three times in PBS and fixed in methanol:acetic acid (3:1) for 20 min at -20°C. After two rinses with fresh fixative, the pellets were dropped onto the slides, air-dried and stored at -20°C until used. Before use, the slides were allowed to stay at room temperature and were rinsed in 2× sodium chloride/sodium citrate (2× SCC, pH 7.0), dehydrated in graded ethanol solutions from 70 to 100%, and air-dried. For identification purposes, the ejaculated cells were stained with Giemsa solution and FISH was performed. The haploid status of the cells was inferred from observation of a single spot for each of the two probes used in FISH. To distinguish spermatocytes of the 1st stage from spermatocytes of the 2nd stage, FISH with subtelomeric probes for chromosomes Xq, Yq, 6q and 14q were used (spermatocytes in stage II revealed a FISH signal from one chromatid). The samples were checked for the presence of leukocytes by the Endtz test [53]. A total of 1,988 cells were analysed in the ejaculates.

### Array CGH and molecular analysis

Genomic DNA was isolated from patients’ peripheral blood leukocytes using Puregene Blood Core Kit B (Qiagen). Control male DNA was purchased from Promega (Madison, WI). Oligonucleotide-based array CGH was performed according to the manufacturer’s protocol (Agilent Technologies, Santa Clara, CA). Restriction digestion and fluorescent labelling were performed using the genomic DNA enzymatic labelling kit (Agilent). The patient samples were labelled with Cyanine-5 dye, and Promega control DNA was labelled with Cyanine-3 dye. The labelled samples were purified with the SureTag DNA labelling kit purification column (Agilent). The yield and probe-specific activity of fluorescent labelling were analysed on a NanoDrop 2000 (Thermo Scientific). The samples were hybridised to SurePrint G3 Human CGH 2x400k Oligo Microarrays (Agilent) for 40 hours at 66°C. The array slides were separated and washed in Agilent Oligo aCGH/ChIP-on-ChIP wash buffers 1 and 2 according to the manufacturer’s protocol (Agilent). The slides were scanned on an Agilent SureScan Microarray C scanner and analysed using Agilent Cytogenomics software. We assessed the quality of DNA gains and losses of all variants using the direct probe signal intensity and the log_2_ ratio of patient/control signals. Following crosscheck, an analysis of the DGV database (Toronto CNV database) revealed the frequency of CNV variants in the normal population.

Molecular analyses of the Y chromosome microdeletions in the AZF region, *SRY* and *CFTR* genes were performed according to methods described by Huleyuk et al [54].

## Competing interests

The authors declare that they have no competing interests.

## Author’s contributions

EW designed the study, drafted the manuscript, collected the results, and analysed and interpreted the data. MO designed the study and collected the results. APG collected the results. NH participated in the collection of the results. BP participated in the data collection. DZ participated in the analysis of the results. SAY participated in the data collection. PJ interpreted the data. ATM interpreted the data. ANY drafted the manuscript and analysed the data. MK designed the study, drafted the manuscript, and analysed and interpreted the data. All authors read and approved the final manuscript.

## Supplementary Material

Additional file 1: Table S1 List of the FISH probes used for characteristics of chromosomes involved in the dic(9;13)(p11.2;p12) (R=red signal, G= green signal and Y= yellow signal). Click here for file

Additional file 2: Table S2 400K genome-wide CGH array results for azoospermic carrier of dic(9;13)(p11.2;p12). Click here for file

Additional file 3: Figure S1 A depiction of a quadrivalent structure. A depiction of a quadrivalent structure in meiotic pachytene formed from the chromosomes involved in the dic(9;13)(p11.2;p12) translocation. Click here for file
