# Supplementary material for: Cytogenetic and molecular analyses of de novo translocation dic(9;13)(p11.2;p12) in an infertile male
Source: Mol Cytogenet. 2014 Feb 21;7:14. doi: 10.1186/1755-8166-7-14 (PMC3944724; doi:10.1186/1755-8166-7-14)
Supplement: Additional file 1: Table S1 — List of the FISH probes used for characteristics of chromosomes involved in the dic(9;13)(p11.2;p12) (R=red signal, G= green signal and Y= yellow signal). [file 1755-8166-7-14-S1.doc]

**Supplementary Table 1** List of the FISH probes used for characteristics of chromosomes involved in the dic(9;13)(p11.2;p12) (R=red signal, G= green signal and Y= yellow signal).

| **Probe**  **No** | **Chromosome** | **Chromosome**  **position** | **Name of marker**  **or RP11 Library No** | **Labeling**  **R=red**  **G=green**  **Y=yellow** | **Figure No** |
| --- | --- | --- | --- | --- | --- |
| 1 | **9** | 9 centromere | *LPE09 | R and/or Y | Fig.2 A, B, D, E |
| 2 | **9** | 9ptel | *LPTP9ptel30 | R | Fig.2 A |
| 3 | **9** | 9p11.2 | RP11-259A5 | R | Fig.2D |
| 4 | **9** | 9p12/9q13 | RP11-318K12 | Y | Fig.2E |
| 5 | **9** | 9qtel | *LPTP9qtel | G | Fig.2 B |
| 6 | **9** | 9 whole paint | *LPP09 | G | Fig.2 C |
| 7 | **13** | 13 whole paint | *LPP13 | R |
| 8 | **13** | 13/21 centromere | **LPE13/21 | G | Fig.2A, D, E |
| 9 | **13** | 13qtel | LPT13 | R | Fig.2 B |
| 10 | **6q** | 6qtel | *LPT06 | R | not shown |
| 11 | **14q** | 14qtel | *LPT14 | G |
| 12 | **15** | 15 centromere | *LPE15 | G | not shown |
| 13 | **18** | 18 centromere | *LPE18 | R |
| 14 | **X** | X centromere | *LPE0X | R and/or G | not shown |
| 15 | **Y** | Y centromere | *LPE0Y | R and/or G |
| 16 | **X/Y** | Xq/Yq | *LPTX,YQ | R or G |
| 17 | **X** | X whole paint | LPP0X | R | Fig.4B |
| 18 | **Y** | Y whole paint | LPP0Y | G |
| 19 | **all** | MultiFISH paint |  |  | Fig.4A |

* FISH probes from Cytocell Technologies LTD., UK

** FISH Vysis probes from Abbot Laboratories, USA
