# Supplementary material for: Cytogenetic and molecular analyses of de novo translocation dic(9;13)(p11.2;p12) in an infertile male
Source: Mol Cytogenet. 2014 Feb 21;7:14. doi: 10.1186/1755-8166-7-14 (PMC3944724; doi:10.1186/1755-8166-7-14)
Supplement: Additional file 2: Table S2 — 400K genome-wide CGH array results for azoospermic carrier of dic(9;13)(p11.2;p12). [file 1755-8166-7-14-S2.doc]

**Supplementary Table 2** 400K genome-wide CGH array results for azoospermic carrier of dic(9;13(p11.2;p12).

| **CNV #** | **Probe**  **Chromosome position** | **Cytoband** | **Probes**  **#** | **Log ratio Amp/Del** | **P-value** | **Gene annotations** | **Toronto DGV freq** | **Gene effect** |
| --- | --- | --- | --- | --- | --- | --- | --- | --- |
| 1 | chr1:72768855-72795480 | p31.1 | 5 | 1,312981 | NA |  | hf pol | benign |
| 2 | chr1:152556449-152586281 | q21.3 | 7 | 1,574342 | NA | *LCE3C* | hf pol | benign |
| 3 | chr1:226814429-226827893 | q42.12 | 3 | 1,572947 | NA | *ITPKB, 3'end* | lf pol | 3'- del |
| 4 | chr2:172961505-172973345 | q31.1 | 4* | 1,408861 | NA | ***DLX2, 11.8 kb whole gene het del*** | not rep | unknown |
| 5 | chr4:3364946-3531614 | p16.3 | 31 | 0,477678 | NA | *RGS12, HGFAC, DOK7...* | hf pol | benign |
| 6 | chr4:69387056-69686275 | q13.2 | 18 | 0,609478 | NA | *UGT2B17, UGT2B15, UGT2B10* | hf pol | benign |
| 7 | chr5:140164398-140266130 | q31.3 | 22 | 0,535172 | NA | *PCDHA1, PCDHA2, PCDHA3...* | hf pol | benign |
| 8 | chr6:32455274-32527058 | p21.32 | 11 | 0,991580 | NA | *HLA-DRB5, HLA-DRB6* | hf pol | benign |
| 9 | chr10:134664199-134697999 | q26.3 | 7 | 1,196016 | NA |  | hf pol | benign |
| 10 | chr12:9637323-9698517 | p13.31 | 8 | 1,656552 | NA |  | hf pol | benign |
| 11 | chr14:19376762-20397731 | q11.2 | 31 | 0,635165 | NA | *OR11H12, POTEG, P704P...* | hf pol | benign |
| 12 | chr14:74001651-74012568 | q24.3 | 3 | 3,191037 | NA | *HEATR4, ACOT1* | hf pol | benign |
| 13 | chr15:20172544-22512790 | q11.1 - q11.2 | 94 | 0,328901 | NA | *GOLGA6L6, GOLGA8C, BCL8...* | hf pol | benign |
| 14 | chr15:22916152-22926015 | q11.2 | 3 | 1,602716 | NA | *CYFIP1* | hf pol | benign |
| 15 | chr16:34472017-34744872 | p11.2 - p11.1 | 22 | 0,659817 | NA | LOC283914, LOC146481 | hf pol | benign |
| 16 | chr17:45497057-45567622 | q21.32 | 7 | 0,923815 | NA | *C17orf57, LOC100272146* | hf pol | benign |
| 17 | chr17:79011647-79054417 | q25.3 | 7 | 0,987245 | NA | *BAIAP2* | hf pol | benign |
| 18 | chr20:1563715-1584485 | p13 | 6 | 2,097298 | NA | *SIRPB1* | hf pol | benign |
| 19 | chr20:14766776-14824431 | p12.1 | 10 | 0,883507 | NA | *MACROD2* | hf pol | benign |
| 20 | chr22:22336268-22553874 | q11.22 | 49 | 0,573224 | NA | *TOP3B* | hf pol | benign |
| 21 | chrX:6456036-6639487 | p22.31 | 16 | 1,000549 | NA |  | hf pol | benign |
| 22 | chrX:8107200-8148580 | p22.31 | 10 | 0,808749 | NA | ***VCX2, whole gene, 1-2 copy gain*** | lf pol | unknown,  testis expressed,  multiple copies |
| 23 | chrX:111465469-111472344 | q23 | 3 | 4,102842 | NA | *ZCCHC16* | hf pol | benign |
| 24 | chrX:154391883-154425684 | q28 | 5 | 2,819552 | NA |  | hf pol | benign |
| 25 | chr16:70155065-70193889 | q21.1 | 5 | 1,196016 | NA | *PDPR, whole gene, 40kb, het del* | hf pol | benign |
| 26 | chr19:13194339-13205350 | q21.1 | 2* | 1,960160 | NA | ***NFIX****,* ***3'end 11kb, homo del,*** | not rep | unknown  low qual |

| red - gain, green -loss |  |  |  |
| --- | --- | --- | --- |
| hf pol - high frequency polymorphic CNV | |  |  |
| lf pol - low frequency polymorphic CNV | |  |  |
| 3'del - 3' end deletion of gene | |  |  |
| * potential low quality due to 2-3 probes showing abnormality | | | |
