# Supplementary figures and images for: Cytogenetic and molecular analyses of de novo translocation dic(9;13)(p11.2;p12) in an infertile male
Source: Mol Cytogenet. 2014 Feb 21;7:14. doi: 10.1186/1755-8166-7-14 (PMC3944724; doi:10.1186/1755-8166-7-14)

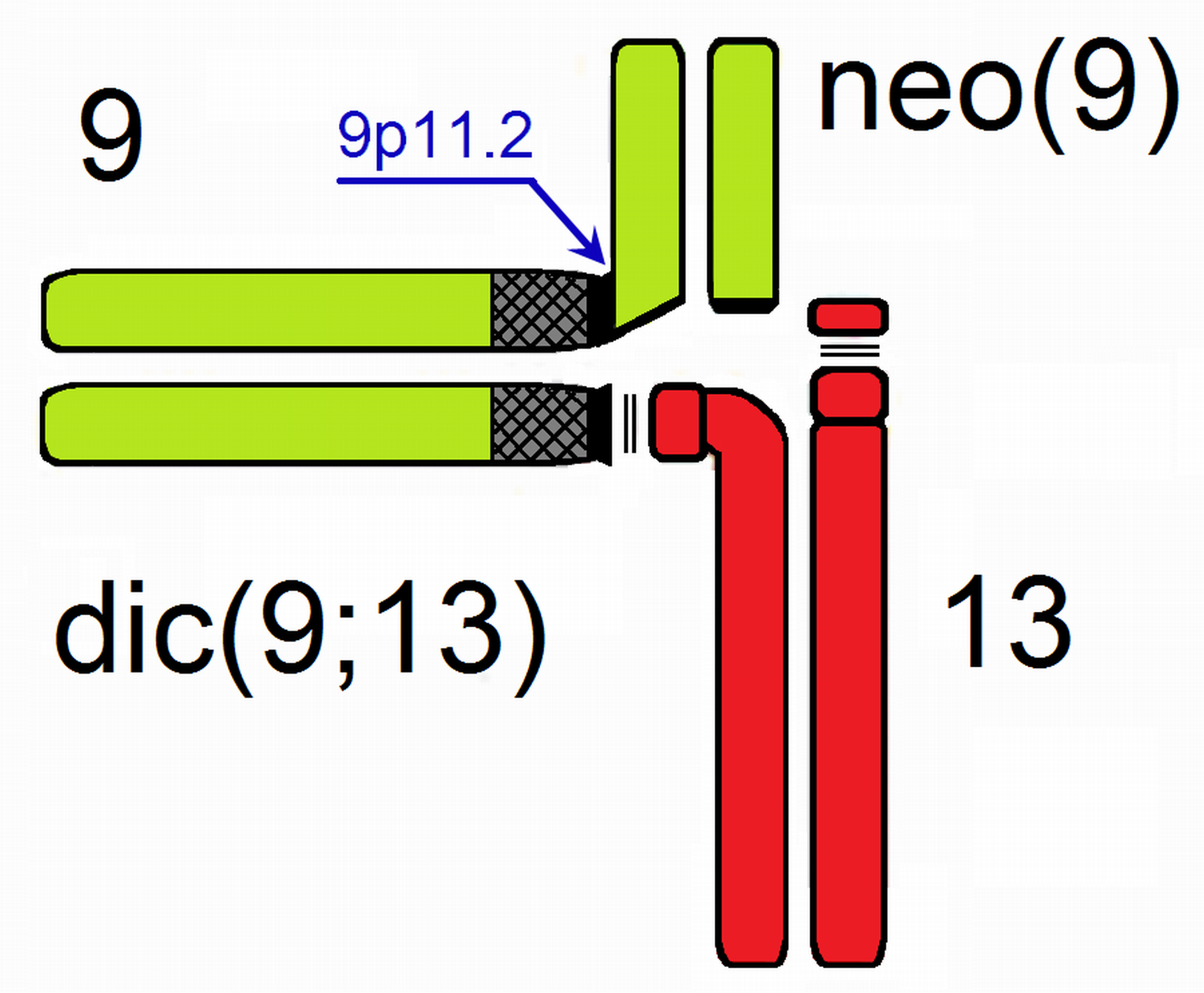

Supplement: Additional file 3: Figure S1 — A depiction of a quadrivalent structure. A depiction of a quadrivalent structure in meiotic pachytene formed from the chromosomes involved in the dic(9;13)(p11.2;p12) translocation. [file 1755-8166-7-14-S3.tiff]
